# Critical values for body mass index related to morbidity in high-volume low-complexity general surgery: a systematic review and meta-analysis

**DOI:** 10.1308/rcsann.2024.0057

**Published:** 2024-08-14

**Authors:** S Hajibandeh, S Hajibandeh, K Harries, WG Lewis, RJ Egan

**Affiliations:** ^1^ Swansea Bay University Health Board, UK; ^2^ University Hospitals of North Midlands NHS Trust, UK; ^3^ Hywel Dda University Health Board, UK

**Keywords:** body mass index; general surgery; cholecystectomy; inguinal hernia; umbilical hernia

## Abstract

**Introduction:**

The aim of this study was to investigate the effect of body mass index (BMI, kg/m^2^) on outcomes of high-volume low-complexity (HVLC) general surgery procedures and to determine critical values for BMI when selecting patients into HVLC programmes.

**Methods:**

A systematic review was conducted of studies looking at patients in different BMI categories undergoing HVLC general surgery procedures (laparoscopic cholecystectomy, inguinal hernia repair and umbilical or paraumbilical hernia repair), in accordance with the PRISMA (Preferred Reporting Items for Systematic reviews and Meta-Analyses) guidelines. A comparison meta-analysis model was constructed to compare the outcomes using random-effects modelling. The QUIPS (Quality In Prognosis Studies) tool and GRADE (Grading of Recommendations Assessment, Development and Evaluation) system were used to assess bias.

**Results:**

A total of 26 studies including 486,392 patients were examined. In laparoscopic cholecystectomy, BMI ≥40 was associated with higher conversion to open surgery (odds ratio [OR]: 1.33, *p*=0.040) but did not affect complications (OR: 0.69, *p*=0.400) or length of hospital stay (mean difference [MD]: −0.01 days, *p*=0.900). In inguinal hernia repair, BMI ≥35 was associated with longer operative time (MD: 18.00 minutes, *p*<0.00001), and higher risk of wound complications (OR: 3.01, *p*<0.00001) and hospital readmission (OR: 1.46, *p*=0.0008). In umbilical or paraumbilical hernia repair, BMI ≥30 was associated with higher risk of wound complications (OR: 6.45, *p*<0.0001) and hospital readmission (OR: 5.56, *p*<0.00001), and longer operative time (MD: 4.01 minutes, *p*=0.030).

**Conclusions:**

Obesity was associated with longer operative time (up to 23 minutes) and higher risk of postoperative morbidity (up to 4-fold) in HVLC procedures. BMI <40 (moderate GRADE certainty – laparoscopic cholecystectomy) and BMI <35 (moderate GRADE certainty – inguinal hernia) appear to represent optimal critical values for perioperative safety metrics.

## Introduction

Recovery of elective care services following the COVID-19 pandemic is a priority for many global healthcare systems.^1–3^ The high-volume low-complexity (HVLC) programme was developed to support the recovery of elective services via standardised pathways, developing surgical hubs and improving theatre productivity.^4^

The HVLC programme recognised general surgery as a key priority area, and focused on laparoscopic cholecystectomy, inguinal hernia repair and umbilical or paraumbilical hernia repair.^4^ Day-case theatre surgery is the default for HVLC procedures, highlighting the importance of proper patient selection to support productivity. In order for patients to be considered for day-case surgery, the procedure must be associated with low risk of blood loss or significant immediate morbidity and it must not require specialist postoperative care; patients must be able to mobilise, and manage pain and oral nutrition at home.^5^

The global obesity epidemic has amplified the number of obese patients undergoing surgical procedures.^6^ In England, 26% of adults are obese and a further 38% are overweight;^7^ similar trends exist in other Western countries.^8^ Obesity and its related comorbidities can adversely affect surgical outcomes,^9,10^ and so it may be reasonable to consider body mass index (BMI, kg/m^2^) as an important patient-related factor when selecting patients for day-case surgery.

The impact of BMI on outcomes following HVLC procedures in general surgery is poorly understood. The aim of this study was to conduct a systematic review and meta-analysis to investigate the effect of BMI on outcomes of HVLC general surgery procedures and to determine critical values for BMI when selecting patients into HVLC programmes.

## Methods

This study was registered on the PROSPERO database (CRD42023477383). It was conducted and reported in accordance with the PRISMA (Preferred Reporting Items for Systematic reviews and Meta-Analyses) statement.^11^

### Eligibility criteria

#### Study design

All observational studies (cohort studies, case controlled studies and case series) were considered for inclusion. Given that BMI is a characteristic rather than an intervention and that it cannot be randomised, identifying a randomised controlled trial on the subject of interest was not likely.

#### Population

All patients aged 18 years or over who underwent laparoscopic cholecystectomy, open or laparoscopic inguinal hernia repair, and open or laparoscopic umbilical or paraumbilical hernia repair were considered eligible for inclusion.

#### Prognostic factor of interest and comparison

Outcomes for patients with a BMI in the following categories were compared for each procedure type:

- BMI ≥30 (obesity) vs BMI 18.5–29.9 (no obesity)
- BMI 25–29.9 (overweight) vs BMI 18.5–24.9 (normal)
- BMI 30–34.9 (class 1 obesity) vs BMI 18.5–24.9 (normal)
- BMI 35–39.9 (class 2 obesity) vs BMI 18.5–24.9 (normal)
- BMI ≥40 (class 3 obesity) vs BMI 18.5–24.9 (normal)

#### Outcomes

Laparoscopic cholecystectomy outcomes included total complications, conversion to open surgery, operative time and length of hospital stay. Hernia repair outcomes included total complications, wound complications, operative time, reoperation and readmission.

### Search methods

A comprehensive search strategy was created by two independent authors with experience in evidence synthesis using proper search limits, keywords, thesaurus headings and operators (Supplementary Table 1). The developed strategy was applied and adopted on the following electronic sources: MEDLINE^®^, the Cumulative Index to Nursing and Allied Health Literature, the Cochrane Central Register of Controlled Trials, Scopus^®^, the International Clinical Trials Registry Platform, the International Standard Randomised Controlled Trial Number Registry, ClinicalTrials.gov and the Grey Literature Network Service. The search strategy had no language restrictions and was last run on 10 October 2023. The reference lists of relevant systematic reviews and original studies were also evaluated to find further eligible studies.

### Study selection and data extraction

The title and abstract of the identified articles were screened, and the full texts of relevant articles were retrieved by two independent authors, who included the studies that met the eligibility criteria. An electronic data collection proforma was evaluated based on randomly selected studies, and included information on name of the first author, year of publication, name of journal, type of study design, description of included population, sample size of each study, BMI categories and outcomes data. The two independent authors discussed and resolved disagreements during study data extraction, and a third independent author was consulted if required.

Two independent authors evaluated the methodological quality of the included studies using the QUIPS (Quality In Prognosis Studies) tool.^12^ This tool evaluates the risk of bias in studies of prognostic factors in terms of study participation, study attrition, prognostic factor measurement, outcome measurement, study confounding, and statistical analysis and reporting.

### Data analysis

RevMan 5.4 software (Nordic Cochrane Centre, Copenhagen, Denmark) was employed for meta-analysis. The odds ratio (OR) and mean difference (MD) were calculated as summary measures for dichotomous and continuous variables respectively. Random-effects modelling was used for analyses and forest plots with 95% confidence intervals (CIs) were constructed to present the results. Individual patients were considered a unit of analysis and where applicable, intention-to-treat information data from the included studies were used for data analysis. Statistical heterogeneity was measured as I^2^ using Cochran's Q test (χ^2^), and heterogeneity was classified as low when I^2^ was 0–25%, moderate when I^2^ was 25–75% and high when I^2^ was 75–100%. Risk of publication bias was evaluated by constructing funnel plots for outcomes reported by at least ten studies.

### Additional analyses

In order to evaluate the consistency and robustness of the results, sensitivity analyses were performed for outcomes reported by a minimum of four studies. Leave-one-out analysis investigated the effect of each study on the pooled outcomes and separate analysis was conducted for studies with low overall risk of bias.

### Certainty of evidence

Assessment of the quality of evidence was based on the recommended standards and domains of the GRADE (Grading of Recommendations Assessment, Development and Evaluation) system.^13^

## Results

**Figure 1:**
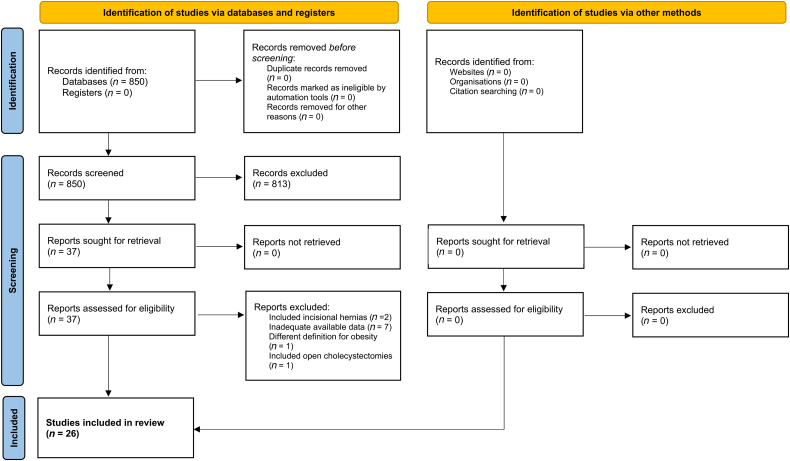
Flowchart of study selection

The search of electronic databases produced 850 articles, of which 813 were excluded directly because they were not relevant to the subject of this review. After reviewing the full text of the remaining 37 articles, 11 more were excluded (7 studies had inadequate available data, 2 studies included patients with incisional hernias, 1 study included open cholecystectomies, 1 study had a different definition for obesity). Consequently, 26 observational studies including a total of 486,392 patients were included.^14–39^ The PRISMA flow diagram is shown in Figure 1 and the baseline characteristics of the included studies are listed in Table 1.

**Table 1:** Baseline characteristics of the included studies

Study	Country	Design	Included population	Sample size	No obesity (BMI <30)	Obesity (BMI ≥30)	Normal (BMI 18.5–24.9)	Overweight (BMI 25–29.9)	Class 1 obesity (BMI 30–34.9)	Class 2 obesity (BMI 35–39.9)	Class 3 obesity (BMI ≥40)
Reeves, 2021^14^	US	Retrospective observational	Patients undergoing laparoscopic cholecystectomy	183,957	90,629	93,328	32,786	57,843	Not reported	Not reported	Not reported
Raakow, 2019^15^	Germany	Retrospective observational	Patients undergoing single-incision laparoscopic cholecystectomy	318	212	106	Not reported	Not reported	Not reported	Not reported	Not reported
Obuchi, 2018^16^	Japan	Retrospective observational	Patients undergoing single-incision laparoscopic cholecystectomy	237	220	17	Not reported	Not reported	Not reported	Not reported	Not reported
Gregori, 2017^17^	Italy	Retrospective observational	Patients undergoing laparoscopic cholecystectomy	730	436	294	Not reported	Not reported	Not reported	Not reported	Not reported
Bowling, 2017^18^	US	Retrospective observational	Patients undergoing laparoscopic cholecystectomy	1,646	900	746	280	620	438	213	95
Wakasugi, 2016^19^	Japan	Retrospective observational	Patients undergoing single-incision laparoscopic cholecystectomy	400	362	38	Not reported	Not reported	Not reported	Not reported	Not reported
Neylan, 2016^20^	US	Retrospective observational	Patients undergoing laparoscopic cholecystectomy	18,228	9,813	8,415	3,871	5,942	4,482	2,200	1,733
Tandon, 2016^21^	UK	Retrospective observational	Patients undergoing laparoscopic cholecystectomy	571	298	273	122	176	Not reported	Not reported	Not reported
Afaneh, 2014^22^	US	Retrospective observational	Patients undergoing laparoscopic cholecystectomy	1,382	903	479	Not reported	Not reported	268	133	78
Yilmaz, 2014^23^	Turkey	Retrospective observational	Patients undergoing single-incision laparoscopic cholecystectomy	202	157	45	Not reported	Not reported	Not reported	Not reported	Not reported
Paajanen, 2012^24^	Finland	Retrospective observational	Patients undergoing laparoscopic cholecystectomy	1,481	1,144	337	Not reported	Not reported	302	135	Not reported
Farkas, 2012^25^	US	Retrospective observational	Patients undergoing laparoscopic cholecystectomy	1,027	536	491	211	325	268	135	88
Chang, 2009^26^	Taiwan	Retrospective observational	Patients undergoing laparoscopic cholecystectomy	627	562	65	310	252	Not reported	Not reported	Not reported
Lee, 2023^27^	US	Retrospective observational	Patient undergoing inguinal hernia repair	161,780	12,8454	33,326	55,917	72,537	25,035	5,901	2,390
Olugbemi, 2023^28^	UK	Retrospective observational	Patient undergoing inguinal hernia repair	185	162	23	69	93	Not reported	Not reported	Not reported
Chinn, 2022^29^	US	Retrospective observational	Patient undergoing robotic inguinal hernia repair	304	222	82	102	120	Not reported	Not reported	Not reported
Elakkad, 2022^30^	Qatar	Retrospective observational	Patient undergoing robotic inguinal hernia repair	99	78	21	78	Not reported	Not reported	Not reported	Not reported
Kudsi, 2022^31^	US	Retrospective observational	Patient undergoing robotic inguinal hernia repair	393	262	131	Not reported	262	Not reported	Not reported	Not reported
Huerta, 2021^32^	US	Retrospective observational	Patient undergoing inguinal hernia repair	600	300	300	Not reported	300	Not reported	Not reported	Not reported
Willoughby, 2017^33^	US	Retrospective observational	Patient undergoing inguinal hernia repair	63,642	51,599	12,043	23,150	28,449	Not reported	Not reported	Not reported
Park, 2011^34^	Korea	Retrospective observational	Patient undergoing inguinal hernia repair	619	443	176	275	168	Not reported	Not reported	Not reported
Rosemar, 2010^35^	Sweden	Retrospective observational	Patient undergoing groin hernia repair	47,374	44,840	2,534	24,987	19,853	Not reported	Not reported	Not reported
Reid, 2009^36^	UK	Retrospective observational	Patient undergoing inguinal hernia repair	125	125	0	35	90	Not reported	Not reported	Not reported
Shahab, 2022^37^	Pakistan	Retrospective observational	Patients undergoing open umbilical hernia repair	150	62	88	Not reported	Not reported	Not reported	Not reported	Not reported
Sadien, 2020^38^	UK	Prospective observational	Patients undergoing open paraumbilical hernia repair	116	78	38	29	49	Not reported	Not reported	Not reported
Yao, 2016^39^	US	Retrospective observational	Patients undergoing open umbilical hernia repair	199	65	134	11	54	73	47	14
BMI = body mass index (kg/m^2^)											

### Risk of bias

**Figure 2:**
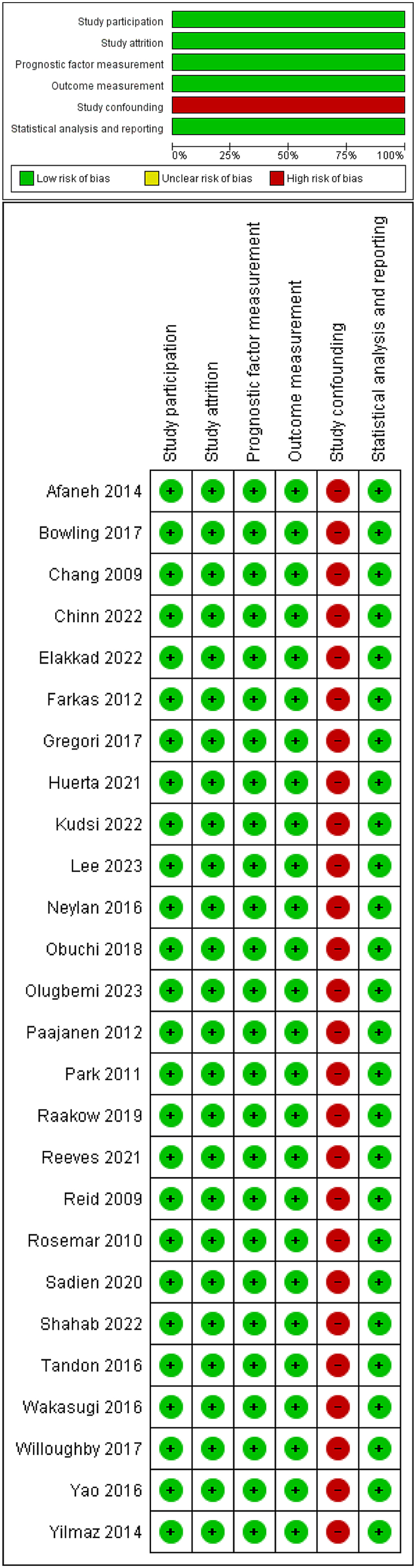
The outcomes of methodological quality assessment of the included studies using the Quality In Prognosis Studies (QUIPS) tool^12^

Figure 2 illustrates the results of the methodological quality assessment based on the QUIPS tool.^12^ All the included studies were judged to be of low risk of bias in terms of study participation, study attrition, prognostic factor measurement, outcome measurement, and statistical analysis and reporting. The included studies were judged to be of high risk of bias in terms of study confounding.

### BMI ≥30 (obese) vs BMI 18.5–29.9 (not obese)

#### Laparoscopic cholecystectomy

Obesity was associated with higher risk of complications (OR: 1.25, 95% CI: 1.06–1.48, *p*=0.009, I^2^=0%, 12 studies, 192,578 patients, GRADE certainty: high) and longer operative time (MD: 9.25 minutes, 95% CI: 6.16–12.33 minutes, *p*<0.00001, I^2^=67%, 8 studies, 5,377 patients, GRADE certainty: high) than no obesity. There was no difference in risk of conversion to open surgery (OR: 1.23, 95% CI: 0.84–1.79, *p*=0.290, I^2^=62%, 12 studies, 26,849 patients, GRADE certainty: high) or length of hospital stay (MD: 0.03 days, 95% CI: −0.09–0.15 days, *p*=0.590, I^2^=0%, 9 studies, 7,320 patients, GRADE certainty: high) (Table 2, Supplementary Figure 1).

**Table 2:** Results of comparative meta-analysis of outcomes between obesity and no obesity in patients undergoing high-volume low-complexity operations

Procedure	Outcome	Number of studies	Number of patients	Summary measure (95% CI)	*p*-value	Heterogeneity	GRADE certainty^13^
Laparoscopic cholecystectomy	Total complications	12	192,578	OR: 1.25 (1.06–1.48)	**0.009**	Low	High
	Conversion to open surgery	12	26,849	OR: 1.23 (0.84–1.79)	0.290	Moderate	High
	Operative time (minutes)	8	5,377	MD: 9.25 (6.16–12.33)	**<0.00001**	Moderate	High
	Length of hospital stay (days)	9	7,320	MD: 0.03 (−0.09–0.15)	0.590	Low	High
Inguinal hernia repair	Total complications	6	273,888	OR: 1.42 (1.10–1.83)	**0.007**	High	Moderate
	Wound complications	7	147,022	OR: 0.52 (0.06–4.73)	0.560	High	Moderate
	Operative time (minutes)	5	163,057	MD: 8.07 (0.90–15.24)	**0.030**	High	Moderate
	Reoperation	6	273,592	OR: 1.09 (0.96–1.25)	0.200	Low	High
	Readmission	3	162,183	OR: 1.06 (0.94–1.19)	0.330	Low	Moderate
Umbilical or paraumbilical hernia repair	Wound complications	1	150	OR: 6.45 (2.75–15.12)	**<0.00001**	–	Very low
	Operative time (minutes)	3	385	MD: 4.01 (0.48–7.54)	**0.030**	High	Very low
	Readmission	1	150	OR: 5.56 (2.71–11.42)	**<0.00001**	–	Very low
CI = confidence interval; GRADE = Grading of Recommendations Assessment, Development and Evaluation; MD = mean difference; OR = odds ratio							

#### Inguinal hernia repair

Obesity was associated with higher risk of total complications (OR: 1.42, 95% CI: 1.10–1.83, *p*=0.007, I^2^=80%, 6 studies, 273,888 patients, GRADE certainty: moderate) and longer operative time (MD: 8.07 minutes, 95% CI: 0.90–15.24 minutes, *p*=0.030, I^2^=98%, 5 studies, 163,057 patients, GRADE certainty: moderate) than no obesity. There was no difference in risk of wound complications (OR: 0.52, 95% CI: 0.06–4.73, *p*=0.560, I^2^=100%, 7 studies, 147,022 patients, GRADE certainty: moderate), reoperation (OR: 1.09, 95% CI: 0.96–1.25, *p*=0.200, I^2^=0%, 6 studies, 273,592 patients, GRADE certainty: high) or hospital readmission (OR: 1.06, 95% CI: 0.94–1.19, *p*=0.330, I^2^=0%, 3 studies, 162,183 patients, GRADE certainty: moderate) (Table 2, Supplementary Figure 2).

#### Umbilical or paraumbilical hernia repair

Obesity was associated with higher risk of wound complications (OR: 6.45, 95% CI: 2.75–15.12, *p*<0.0001, I^2^=not applicable, 1 study, 150 patients, GRADE certainty: very low) and readmission (OR: 5.56, 95% CI: 2.71–11.42, *p*<0.00001, I^2^=not applicable, 1 study, 150 patients, GRADE certainty: very low), and with longer operative time (MD: 4.01 minutes, 95% CI: 0.48–7.54 minutes, *p*=0.030, I^2^=94%, 3 studies, 385 patients, GRADE certainty: very low) than no obesity (Table 2, Supplementary Figure 3). The other outcomes for this comparison were not reported by the included studies.

### Variation in the risk of outcomes among different BMI categories

The variations in the pooled risk of each outcome among different BMI categories for each procedure are demonstrated visually in Figure 3 and are described in detail below.

**Figure 3:**
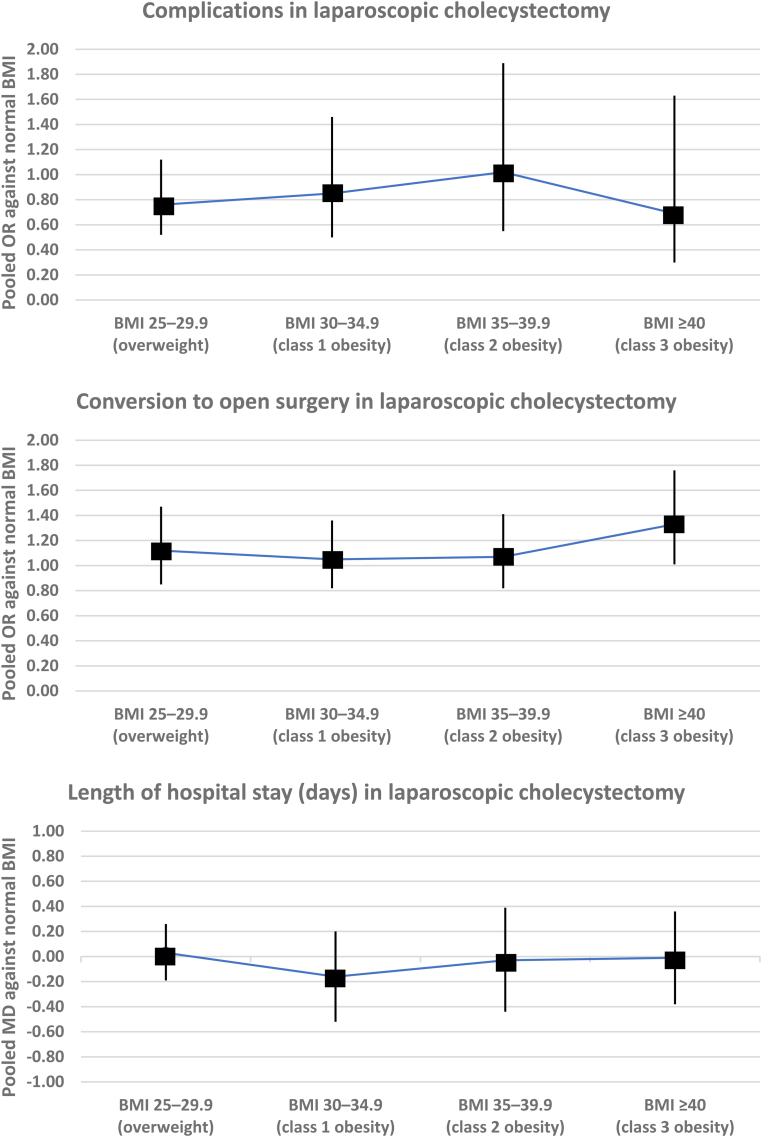
Variations in the pooled summary measures (odds ratio [OR] and mean difference [MD]) stratified based on different body mass index (BMI, kg/m^2^) categories (*Continued.*)

**Figure 3:**
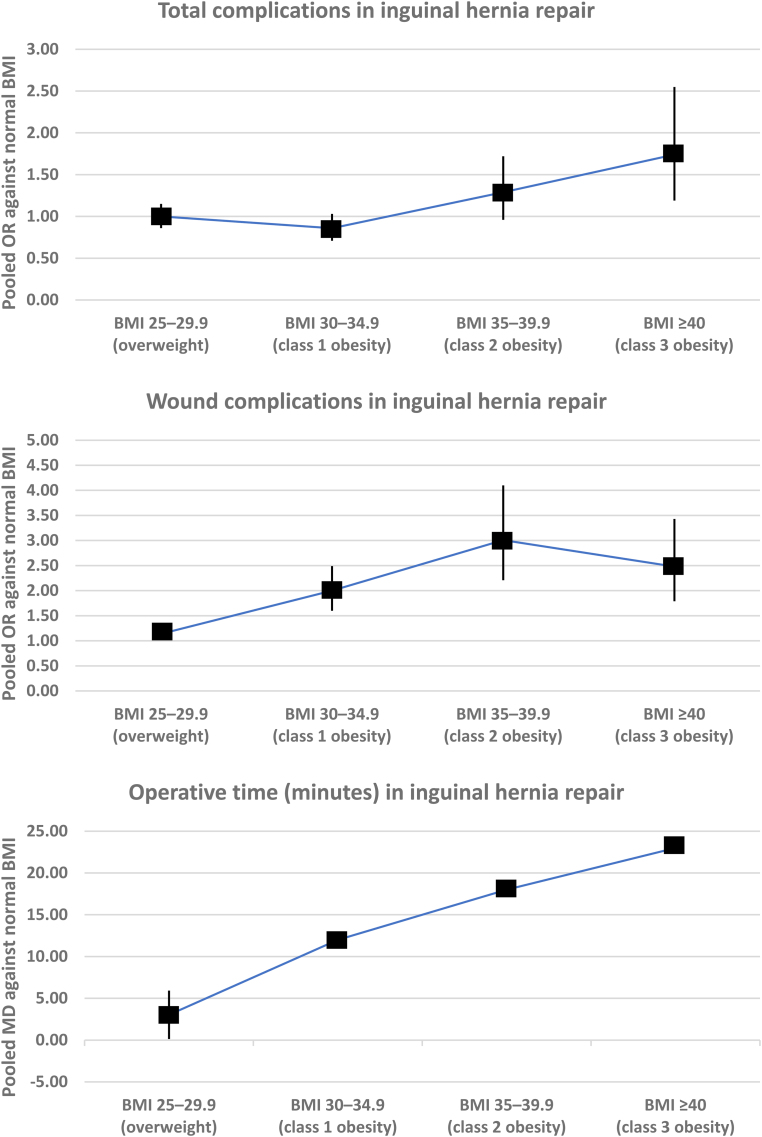
Continued.

**Figure 3:**
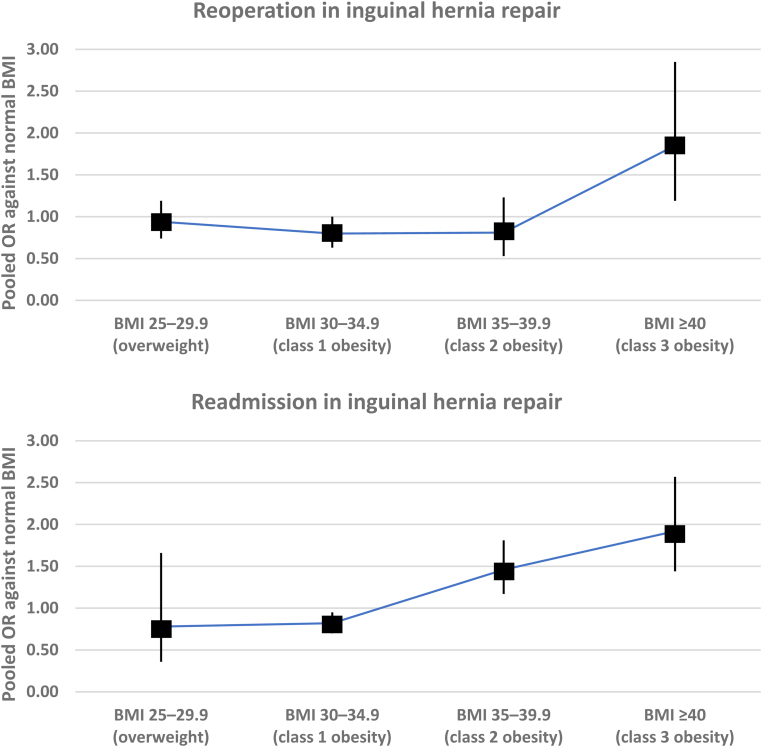
Continued.

#### BMI 25–29.9 (overweight) vs BMI 18.5–24.9 (normal)

*Laparoscopic cholecystectomy*: There was no difference in risk of complications (OR: 0.76, 95% CI: 0.52–1.12, *p*=0.160, I^2^=24%, 5 studies, 92,925 patients, GRADE certainty: high) or conversion to open surgery (OR: 1.12, 95% CI: 0.85–1.47, *p*=0.430, I^2^=11%, 5 studies, 12,109 patients, GRADE certainty: high), or in length of hospital stay (MD: 0.03 days, 95% CI: −0.19–0.26 days, *p*=0.790, I^2^=0%, 3 studies, 1,998 patients, GRADE certainty: moderate) between BMI 25–29.9 and normal BMI. BMI 25–29.9 was associated with longer operative time (MD: 10.50 minutes, 95% CI: 3.96–17.04 minutes, *p*<0.00001, I^2^=not applicable, 1 study, 562 patients, GRADE certainty: moderate) than normal BMI (Supplementary Figure 1).

*Inguinal hernia repair*: BMI 25–29.9 was associated with higher risk of wound complications (OR: 1.14, 95% CI: 1.02–1.29, *p*=0.030, I^2^=18%, 6 studies, 225,859 patients, GRADE certainty: high) and longer operative time (MD: 3.03 minutes, 95% CI: 0.14–5.92 minutes, *p*=0.040, I^2^=78%, 3 studies, 128,801 patients, GRADE certainty: moderate) than normal BMI. There was no difference in risk of total complications (OR: 1.00, 95% CI: 0.86–1.15, *p*=0.960, I^2^=68%, 4 studies, 225,512 patients, GRADE certainty: high), reoperation (OR: 0.94, 95% CI: 0.74–1.19, *p*=0.600, I^2^=73%, 4 studies, 225,115 patients, GRADE certainty: high) or readmission (OR: 0.78, 95% CI: 0.36–1.66, *p*=0.510, I^2^=16%, 3 studies, 128,801 patients, GRADE certainty: moderate) (Supplementary Figure 2).

*Umbilical or paraumbilical hernia repair*: There was no difference in operative time (MD: −3.17 minutes, 95% CI: −13.54–7.20 minutes, *p*=0.550, I^2^=85%, 2 studies, 143 patients, GRADE certainty: very low) (Supplementary Figure 3). The other outcomes for this comparison were not reported.

#### BMI 30–34.9 (class 1 obesity) vs BMI 18.5–24.9 (normal)

*Laparoscopic cholecystectomy*: There was no difference in risk of complications (OR: 0.85, 95% CI: 0.50***–***1.46, *p*=0.560, I^2^=0%, 2 studies, 1,197 patients, GRADE certainty: moderate) or conversion to open surgery (OR: 1.05, 95% CI: 0.82***–***1.36, *p*=0.680, I^2^=2%, 3 studies, 9,550 patients, GRADE certainty: moderate), or in length of hospital stay (MD: −0.16 days, 95% CI: −0.52–0.20 days, *p*=0.390, I^2^=34%, 2 studies, 1,197 patients, GRADE certainty: moderate) between BMI 30−34.9 and normal BMI. Operative time for this comparison was not reported (Supplementary Figure 1).

*Inguinal hernia repair*: BMI 30−34.9 was associated with higher risk of wound complications (OR: 2.00, 95% CI: (1.60−2.49, *p*<0.00001, I^2^=not applicable, 1 study, 80,952 patients, GRADE certainty: moderate) and longer operative time (MD: 12.00 minutes, 95% CI: 11.84–12.16 minutes, *p*<0.00001, I^2^=not applicable, 1 study, 80,952 patients, GRADE certainty: moderate) than normal BMI. There was no difference in risk of total complications (OR: 0.86, 95% CI: 0.71−1.03, *p*=0.110, I^2^=not applicable, 1 study, 80,952 patients, GRADE certainty: moderate) or reoperation (OR: 0.80, 95% CI: 0.80−1.00, *p*=0.050, I^2^=not applicable, 1 study, 80,952 patients, GRADE certainty: moderate). BMI 30−34.9 was associated with lower risk of hospital readmission (OR: 0.82, 95% CI: 0.70−0.95, *p*=0.009, I^2^=not applicable, 1 study, 80,952 patients, GRADE certainty: moderate) than normal BMI (Supplementary Figure 2).

*Umbilical or paraumbilical hernia repair*: The outcomes for this comparison were not reported.

#### BMI 35−39.9 (class 2 obesity) vs BMI 18.5−24.9 (normal)

*Laparoscopic cholecystectomy*: There was no difference in risk of complications (OR: 1.02, 95% CI: 0.55−1.89, *p*=0.960, I^2^=0%, 2 studies, 839 patients, GRADE certainty: moderate) or conversion to open surgery (OR: 1.07, 95% CI: 0.82−1.41, *p*=0.610, I^2^=0%, 3 studies, 6,910 patients, GRADE certainty: moderate), or in length of hospital stay (MD: −0.03 days, 95% CI: −0.44–0.39 days, *p*=0.900, I^2^=35%, 2 studies, 839 patients, GRADE certainty: moderate) between BMI 35–39.9 and normal BMI. Operative time for this comparison was not reported (Supplementary Figure 1).

*Inguinal hernia repair*: BMI 35–39.9 was associated with higher risk of wound complications (OR: 3.01, 95% CI: 2.21–4.10, *p*<0.00001, I^2^=not applicable, 1 study, 61,818 patients, GRADE certainty: moderate) and readmission (OR: 1.46, 95% CI: 1.17–1.81, *p*=0.0008, I^2^=not applicable, 1 study, 61,818 patients, GRADE certainty: moderate), and with longer operative time (MD: 18.00 minutes, 95% CI: 17.66–18.34 minutes, *p*<0.00001, I^2^=not applicable, 1 study, 61,818 patients, GRADE certainty: moderate) than normal BMI. There was no difference in risk of total complications (OR: 1.29, 95% CI: 0.96–1.72, *p*=0.090, I^2^=not applicable, 1 study, 61,818 patients, GRADE certainty: moderate) or reoperation (OR: 0.81, 95% CI: 0.53–1.23, *p*=0.330, I^2^=not applicable, 1 study, 61,818 patients, GRADE certainty: moderate) (Supplementary Figure 2).

*Umbilical or paraumbilical hernia repair*: The outcomes for this comparison were not reported.

#### BMI ≥40 (class 3 obesity) vs BMI 18.5–24.9 (normal)

*Laparoscopic cholecystectomy*: There was no difference in risk of complications (OR: 0.69, 95% CI: 0.30–1.63, *p*=0.400, I^2^=0%, 2 studies, 674 patients, GRADE certainty: moderate) or length of hospital stay (MD: –0.01 days, 95% CI: –0.38–0.36 days, *p*=0.900, I^2^=0%, 2 studies, 674 patients, GRADE certainty: moderate) between BMI ≥40 and normal BMI. BMI ≥40 was associated with higher risk of conversion to open surgery (OR: 1.33, 95% CI: 1.01–1.76, *p*=0.040, I^2^=0%, 3 studies, 6,278 patients, GRADE certainty: moderate) than normal BMI. Operative time for this comparison was not reported (Supplementary Figure 1).

*Inguinal hernia repair*: BMI ≥40 was associated with higher risk of risk of total complications (OR: 1.74, 95% CI: 1.19–2.55, *p*=0.004, I^2^=not applicable, 1 study, 58,307 patients, GRADE certainty: moderate), wound complications (OR: 2.48, 95% CI: 1.79–3.43) *p*<0.00001, I^2^=not applicable, 1 study, 58,307 patients, GRADE certainty: moderate), reoperation (OR: 1.85, 95% CI: 1.19–2.85, *p*=0.006, I^2^=not applicable, 1 study, 58,307 patients, GRADE certainty: moderate) and readmission (OR: 1.92, 95% CI: 1.44–2.57, *p*<0.00001, I^2^=not applicable, 1 study, 58,307 patients, GRADE certainty: moderate), and with longer operative time (MD: 23.00 minutes, 95% CI: 22.38–23.62 minutes, *p*<0.00001, I^2^=not applicable, 1 study, 58,307 patients, GRADE certainty: moderate) than normal BMI (Supplementary Figure 2).

*Umbilical or paraumbilical hernia repair*: The outcomes for this comparison were not reported.

### Sensitivity analyses

Sensitivity analyses confirmed consistency of the results. Leave-one-out analysis showed that removal of one study at a time did not affect the pooled risk of any of the outcomes. Moreover, separate analysis of studies with low overall risk of bias did not affect the pooled risk of any of the outcomes.

### Risk of publication bias

The risks of complications and conversion to open surgery in patients undergoing laparoscopic cholecystectomy were reported by more than ten studies. The visual assessment of funnel plots suggested that the risk of publication bias was low (Supplementary Figure 4).

## Discussion

HVLC surgery is generally considered safe and routine. Nevertheless, the safety and success of any surgical procedure depends on a variety of factors, including the patient's overall health, the surgical team's expertise, and the availability of the necessary resources and equipment. In addition, individual patient factors may influence the perceived complexity and risk associated with any given procedure.

The principal findings of this systematic review and meta-analysis of 486,392 patients from 26 observational studies showed that obesity (BMI ≥30) was associated with longer operative time (up to 23 minutes) and higher risk of postoperative morbidity (up to 4-fold) in patients undergoing HVLC general surgery procedures. BMI ≥40 (moderate GRADE certainty) resulted in a 1.3-fold higher risk of conversion to open surgery during laparoscopic cholecystectomy, BMI ≥35 (moderate GRADE certainty) in a threefold higher risk of wound complications as well as longer operative time (up to 18 minutes) in inguinal hernia repair, and BMI ≥30 (very low GRADE certainty) in a fourfold higher risk of wound complications in umbilical and paraumbilical hernia repair. Sensitivity analyses confirmed the robust nature of the results.

This is the first systematic review of the influence of BMI categories on outcomes of HVLC general surgery and consequently, at the time of writing, the evidence base with which to compare the findings of this study is thin. However, the findings are in keeping with the available literature.

Christina and Wijaya conducted a systematic review of nine studies (7,138 patients) and reported that related to laparoscopic cholecystectomy, obesity was associated with a higher risk of conversion to open surgery and a longer operative time.^40^ Although their study did not compare the outcomes between different BMI categories, their findings corroborate the findings of the current study.

Moreover, Sarkhosh *et al* conducted a narrative systematic review of ten studies (70,730 patients) and reported a trend towards higher risk of complications and longer hospital stay following inguinal hernia repair.^41^ Sarkhosh *et al* lacked formal meta-analysis and did not report outcomes related to specific BMI categories. In addition, the length of stay reported in all patient groups in their study far exceeded the current aim to perform a high proportion of inguinal hernia repairs in day-case units.^42,43^

The available literature on the effect of different BMI categories on outcomes of umbilical or paraumbilical hernia repair is even more limited. Nevertheless, the findings of our study are consistent with those reported in the setting of abdominal wall ventral hernia repair.^44,45^

Our study has inherent limitations. The retrospective nature of most of the included studies introduces the inevitable risk of selection and confounding bias. Given the limited number of studies related to umbilical or paraumbilical hernia repair, any conclusions regarding outcomes of this type of herniorrhaphy are subject to type 2 statistical error. It was not possible to access data from individual patients from the included studies and receiver operating characteristic curve analysis to evaluate critical cut-off values for BMI for the outcomes could therefore not be performed.

The findings of the current study may be useful for developing standardised pathways as part of a planned HVLC programme, based on day-case surgery, which includes procedures with reasonably short operative time and low risk of immediate complications.^4,5^ Considering the global obesity epidemic,^6^ it is common to encounter patients with different classes of obesity on elective surgical waiting lists and the available pathways lack objective data to inform decisions on the risks associated with specific patients' BMIs.

The results of the current study suggest that in patients undergoing laparoscopic cholecystectomy, class 3 BMI (≥40, severe obesity) may adversely influence HVLC programmes by increasing the risk of conversion to open surgery and prolonging operative time, resulting in conversion of a day-case procedure to inpatient stay. On the other hand, in patients undergoing inguinal hernia repair, class 2 BMI (≥35) may adversely influence HVLC programmes by prolonging operative time, and increasing the risks of wound complications and hospital readmissions.

## Conclusions

Obesity is associated with longer operative time (up to 23 minutes) and higher risk of postoperative morbidity (up to 4-fold) in patients undergoing HVLC procedures in general surgery, with accompanying costs in terms of theatre time and postoperative medical resources related to complication treatment, hospital readmission, convalescence and return to work. Based on the available evidence, any reasonable observer may accept BMI <40 (moderate GRADE certainty) for laparoscopic cholecystectomy and BMI <35 (moderate GRADE certainty) for inguinal hernia repair as being evidence-based critical values when selecting patients into HVLC programmes.
